# Leveraging artificial intelligence for mycotoxin management in food systems

**DOI:** 10.1038/s41538-026-01005-0

**Published:** 2026-07-30

**Authors:** Francesca Caloni, Thomas Hartung

**Affiliations:** 1 Department of Environmental Science and Policy (ESP), Università degli Studi di Milano, Milan, Italy; 2 Doerenkamp-Zbinden Chair for Evidence-Based Toxicology, Johns Hopkins Bloomberg School of Public Health and Whiting School of Engineering, Baltimore, MD USA; 3 University of Konstanz, Konstanz, Germany

**Keywords:** Risk factors, Agriculture

## Abstract

AI-driven methods have been applied across mycotoxin research in detection, prediction, and control, offering high laboratory accuracy ( > 90%), robust forecasting (75–99%), and emerging mitigation strategies (80–86%). Integrating AI with multi-omics data, mixture toxicity modeling, and standardized protocols promises to enhance food safety, streamline regulatory decision-making, and reduce animal testing.

## Introduction

Mycotoxins—secondary metabolites produced by filamentous fungi—pose ubiquitous threats to global food security, public health, and agricultural economies. Common mycotoxins such as aflatoxins, ochratoxins, fumonisins, trichothecenes, and zearalenone are regulated internationally due to their acute toxicity, carcinogenicity, immunosuppressive, and hepatotoxic effects^1^. Emerging mycotoxins, including enniatins and beauvericin, lack comprehensive regulatory frameworks yet are increasingly detected in diverse commodities, including infant foods and cheese^2^. More than 400 mycotoxins have been suggested, with only 30 known to be harmful to humans and animals^3^, which challenges traditional control mechanisms.

Traditional mycotoxin analysis relies on chromatography (HPLC, GC-MS, LC-MS) and immunoassays (ELISA), which, while sensitive, demand extensive sample preparation, specialized equipment, and expert interpretation, resulting in high costs and delayed turnaround times^4^. These limitations constrain large-scale surveillance and timely risk mitigation in supply chains.

Artificial Intelligence (AI) encompasses machine learning (ML), deep learning (DL), and related computational techniques that can learn complex patterns from large datasets. AI approaches, compared to conventional regression or mechanistic models, offer enhanced capacity to integrate multi-modal datasets, capture non-linear interactions, and adapt dynamically as new data becomes available, making them a powerful complement rather than a replacement for existing tools. Unlike previous reviews that focus on specific analytical techniques, this Perspective uniquely synthesizes findings across the entire mycotoxin management continuum—from detection to prediction to mitigation—highlighting how AI can transform the field by enabling more integrated approaches to food safety. To identify relevant publications, we used an AI-assisted screening strategy; the search protocol and a longitudinal evaluation of Elicit consistency across repeated runs are described in the Supplementary Information. Because repeated Elicit runs differed in included studies and reporting quality, all evidence discussed here was manually curated before inclusion.

In the following sections, we examine how AI is revolutionizing mycotoxin detection, forecasting contamination risks, informing mitigation strategies, and enabling next-generation regulatory frameworks. We conclude by outlining a roadmap for realizing AI’s full potential in safeguarding food systems against mycotoxin threats.

## AI-driven detection and classification

### Conventional vs AI-enhanced detection

**Fig. 1:**
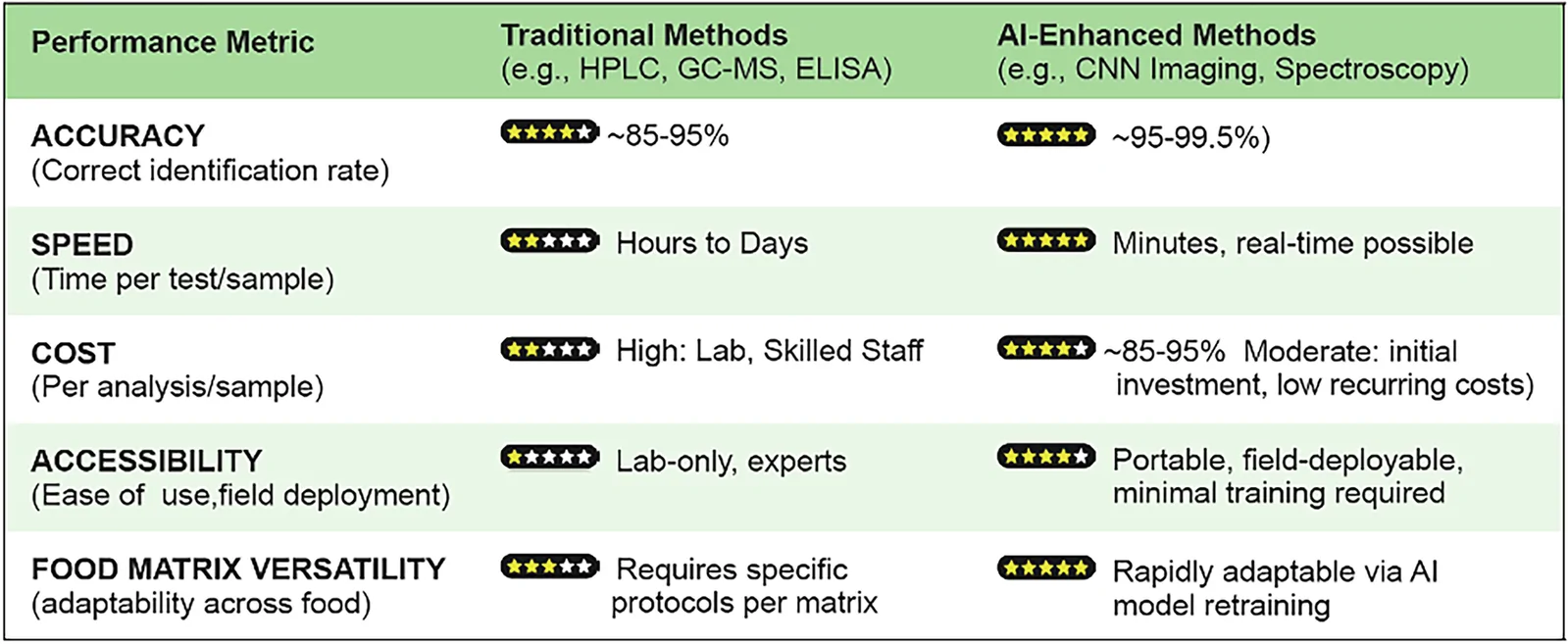
Performance comparison of traditional vs. AI-enhanced mycotoxin detection methods. Traditional methods (chromatography/immunoassays) are highly accurate but slow, expensive, require skilled personnel, and are limited to laboratory settings. AI-enhanced methods offer superior accuracy, significantly faster detection speeds, cost-effectiveness after initial setup, ease of use, and excellent adaptability across diverse food matrices due to retrainable AI models. ★★★★★ Excellent, ★★★★**☆** Very Good, ★★★**☆☆** Moderate, ★★**☆☆☆** Limited, ★**☆☆☆☆** Poor.

Traditional analytical methods, such as LC-MS and ELISA, provide highly accurate quantitative mycotoxin measurements and, in many cases (e.g., LC-MS/MS), can be scaled for high-throughput analysis in well-equipped laboratories. However, these still require centralized facilities, skilled operators, and significant processing time, which limits their utility for rapid, field-deployable screening. AI-enhanced approaches are transforming this landscape by enabling rapid, non-destructive analysis through algorithm integration with imaging and spectroscopic platforms^5–7^. These innovations represent a paradigm shift from lab-confined testing to deployable field solutions (Fig. 1).

### Advanced algorithms and performance patterns

A systematic assessment of 18 AI-based detection studies (supplement) revealed remarkable performance across diverse algorithms^3,8,9^. Deep learning models, particularly convolutional neural networks, demonstrate exceptional capability in image-based detection, with accuracy reaching 99.5% when applied to visible and near-infrared imaging of contaminated crops^6,9^. Support vector machines and ensemble methods like XGBoost have shown similar promise when paired with spectroscopic data, achieving detection accuracies of 95–100% across various mycotoxins and matrices^7,8,10^.

The integration of AI with novel sensing modalities—such as electronic noses that detect volatile biomarkers of contamination—is particularly promising for field deployment, with classification accuracies exceeding 98%^5^. These technologies could democratize testing by reducing reliance on centralized laboratories.

### Bridging laboratory excellence and field reality

Despite impressive laboratory performance, translating AI detection systems to real-world settings remains challenging. Standardized protocols for data acquisition, robust cross-platform calibration methods, and validation across diverse environmental conditions are essential prerequisites for widespread adoption^5^. Industry-academia partnerships focused on creating open datasets and benchmarking standards would significantly accelerate progress in this domain.

## Predictive modeling for mycotoxin occurrence

### The forecasting imperative

Predicting mycotoxin contamination before it occurs represents perhaps AI’s most transformative potential in food safety. By integrating environmental, agronomic, and storage data, AI models can forecast contamination risks and enable proactive interventions that prevent—rather than merely detect—food safety threats.

### Algorithmic approaches and their promise

Our analysis identified seven algorithmic approaches being applied to mycotoxin forecasting, each with distinctive strengths^5,8^. Deep neural networks and gradient boosting machines have demonstrated particular promise for integrating complex environmental and agronomic factors, achieving prediction accuracies exceeding 75% and 96%, respectively^5,11^. For example, Liu et al. ^11^ applied gradient boosting to meteorological and agronomic datasets to predict deoxynivalenol (DON) levels in European wheat, achieving an AUC of 0.92 in external validation. Bayesian networks offer the additional advantage of interpretability, crucial for stakeholder trust, while maintaining robust performance ( > 94% accuracy)^5^.

The most promising direction appears to be hybrid models that combine mechanistic understanding of fungal biology with the pattern-recognition capabilities of machine learning. These approaches not only offer strong predictive performance but also provide insights into the causal factors driving contamination—knowledge that can inform long-term mitigation strategies.

### Toward operational early warning systems

The true value of predictive models lies in their integration into decision support systems that deliver actionable intelligence to stakeholders. User-friendly dashboards and mobile applications can translate complex model outputs into practical recommendations, such as adjusting harvest timing or modifying storage conditions. Pilot implementations of integrated decision support systems for mycotoxin management in feed and food supply chains demonstrate the feasibility of this approach for operationalizing predictive model outputs^12^.

## Mitigation and control strategies

### AI-guided intervention planning

**Fig. 2:**
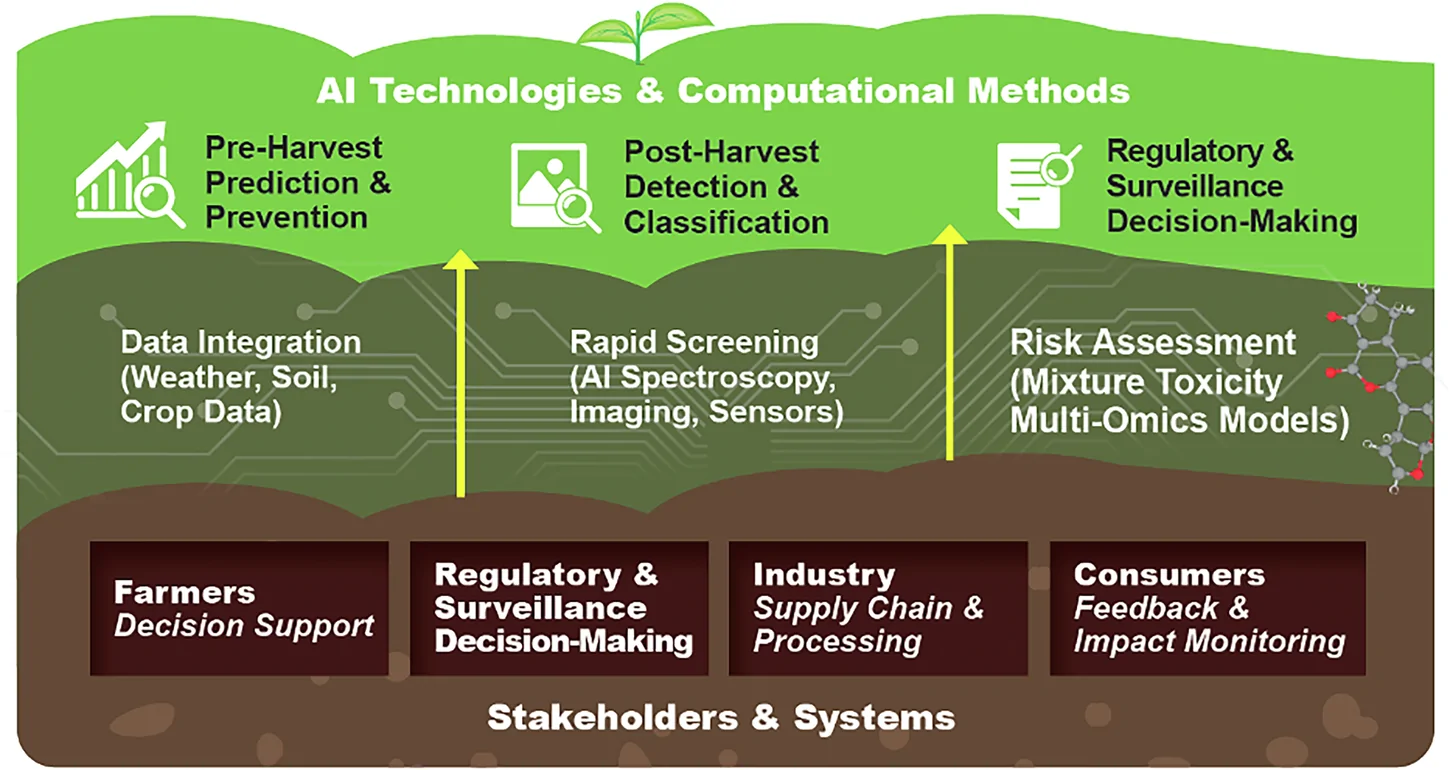
Conceptual framework for AI integration in mycotoxin management. Pre-Harvest Prediction & Prevention: AI integrates environmental data (weather, soil moisture), crop monitoring, and historical data to forecast mycotoxin risks and recommend preventive actions. Post-Harvest Detection & Classification: Advanced AI-driven spectroscopic and imaging techniques rapidly identify and classify contaminated crops with high accuracy, enabling swift action to manage contaminated batches. Regulatory & Surveillance Decision-Making: AI-informed regulatory frameworks use multi-omics and mixture toxicity models to ensure holistic, precise risk assessments, streamlining oversight and response actions. Stakeholders & Systems Integration: AI-enhanced decision support systems enable actionable insights for farmers, industry players, regulators, and consumers. Real-time data flows among these stakeholders, facilitated by blockchain and interoperable data systems, enhance traceability, accountability, and responsive management. This framework emphasizes interconnected data flows and cooperative engagement among stakeholders, underlining the transformative potential of AI in ensuring safer food systems.

While detection and prediction have received more attention, AI applications in mycotoxin control (Fig. 2) show significant promise. Decision support systems combining random forests and Bayesian networks have demonstrated 80–86% accuracy in recommending optimal intervention strategies, including biocontrol agent application timing and post-harvest processing adjustments^12^. Computer vision-based sorting systems have similarly proven effective for removing contaminated kernels from grain batches.

### Biological solutions and smart infrastructure

AI is accelerating biological approaches to mycotoxin management through intelligent screening of microbial antagonists^13,14^ and optimization of enzymatic detoxification processes^15–17^. These approaches offer potentially more sustainable alternatives to chemical treatments. For example, deep learning models have been applied to screen Bacillus and Trichoderma strains for their inhibitory potential against mycotoxigenic fungi, and AI-guided enzyme engineering has optimized laccase variants for enhanced degradation of aflatoxin B1. Additionally, emerging Internet of Things (IoT) platforms integrated with AI analytics enable continuous monitoring of storage conditions, automatically triggering preventive measures when conditions favor mycotoxin development^5^.

### Blockchain-enhanced traceability

The integration of blockchain technology with AI monitoring systems represents a particularly promising frontier for mycotoxin management. These systems can create immutable records of environmental conditions and testing results throughout the supply chain, enhancing accountability and enabling rapid response to contamination events^18^. Early implementations in coffee and grain supply chains demonstrate the feasibility of this approach for high-value commodities^18^^,19^.

### Future innovations on the horizon

Looking ahead, we anticipate several breakthrough technologies that could revolutionize mycotoxin control. These include CRISPR-engineered microbiomes that selectively inhibit mycotoxigenic fungi, AI-optimized bioactive packaging materials that respond to contamination signals, and distributed sensor networks that enable community-based monitoring in smallholder farming contexts.

## Integration into food safety protocols

### Standardization and interoperability

For AI tools to achieve mainstream adoption in food safety systems, they must integrate seamlessly with existing protocols and regulatory frameworks. This requires development of consistent data formats, standardized ontologies for mycotoxin metadata, and application programming interfaces (APIs) that connect AI models with laboratory information management systems^5,12^.

### Economic considerations and implementation pathways

The economic calculus of AI adoption warrants careful consideration. Initial implementation costs for AI systems can be substantial, yet potential returns through reduced testing costs, decreased food waste, and prevented contamination events are significant. Although cost–benefit studies are scarce, analogous cases of risk-management co-regulation (e.g., aflatoxin in maize) show multi-million-dollar annual gains, while broader AI-based food safety systems often achieve ROI within 12–24 months in industrial settings. This suggests that in high-risk commodity chains with favorable adoption contexts, AI deployment may recover costs within 2–3 growing seasons—even for medium-sized operations.

Implementation pathways will necessarily vary by region and commodity chain. Large-scale processors and multinational exporters are likely early adopters, while cooperative models may facilitate access for smaller producers. Public-private partnerships that subsidize initial system deployment could accelerate adoption in regions particularly vulnerable to mycotoxin contamination.

### Regulatory innovation and harmonization

Regulatory frameworks must evolve to accommodate AI-driven approaches to mycotoxin management. Case studies from Italy and Brazil described in recent reviews demonstrate how AI-driven prediction models can be applied to national datasets, illustrating their potential to enhance mycotoxin surveillance and support more responsive food safety oversight^20^. International harmonization of validation criteria for AI tools would further accelerate adoption by reducing regulatory uncertainties.

### Deployment in low- and middle-income countries (LMICs)

Given the disproportionate mycotoxin burden in LMICs, AI-based solutions must address limited digital infrastructure, lack of open datasets, and cost constraints. Cooperative models, mobile-based detection platforms, and donor-supported pilot programs can help bridge these gaps and ensure equitable access to AI-enabled food safety.

## Challenges and future directions

### Data limitations and quality concerns

The development of robust AI models is hindered by data scarcity, particularly for emerging mycotoxins and understudied commodity chains. International initiatives to create open, annotated datasets could address this fundamental constraint^8,12,20^. A significant proportion of studies did not report complete dataset characteristics, which hinders reproducibility and meta-analysis.

### Explainability and trust

Complex models, particularly deep neural networks, often function as “black boxes,” limiting regulatory acceptance and stakeholder trust. Explainable AI (xAI) methods that illuminate feature importance and decision pathways are essential for building confidence in AI-driven food safety systems^21,22^. Visualization tools that communicate model reasoning in intuitive ways could bridge the gap between technical performance and practical acceptance.

### Identification of novel health hazards and relevant mycotoxins

While more than 400 putative mycotoxins have been identified, their isolation or synthesis in sufficient amounts for traditional toxicity testing is impossible. For example, a single rat bioassay for carcinogenicity can require up to 10 kg of test material in order to treat 400 rats every day for two years with maximum tolerated doses. AI lends itself to the identification of such health risks^21–23^. Studies to apply AI-based predictive toxicity to mycotoxins are on the way, which could help identify novel relevant mycotoxins and unexpected health effects.

### Beyond single compounds: modeling mycotoxin mixtures

Traditional risk assessment approaches that focus on individual mycotoxins fail to capture the complex reality of co-occurring contaminants. AI offers unprecedented capabilities to model synergistic and antagonistic interactions in mycotoxin mixtures, leveraging deep learning to predict additive toxicity from high-dimensional dose-response data^21–23^. These models could transform regulatory approaches, moving from single-compound limits to more holistic safety assessments.

### Multi-omics integration: toward precision toxicology

The integration of genomic, transcriptomic, proteomic, and metabolomic data with AI models represents perhaps the most exciting frontier in mycotoxin research. This approach could uncover novel biomarkers of exposure and effect, enabling physiologically based toxicokinetic modeling without reliance on animal testing^21,23^. Such advances would not only enhance safety assessment but also align with global efforts to reduce animal experimentation.

### Building human capacity

The successful implementation of AI in mycotoxin management ultimately depends on human expertise. Training programs for food safety professionals, farmers, and regulators must go beyond technical skills to foster critical understanding of AI capabilities and limitations. Participatory design approaches that engage end-users throughout development can ensure that AI tools address genuine needs rather than technology-driven agendas.

## Conclusion

AI technologies are poised to transform mycotoxin management across the food system, from farm to fork. High-accuracy detection systems, robust forecasting models, and emerging decision support tools illustrate AI’s potential to enhance food safety, reduce economic losses, and protect public health. Realizing this potential requires addressing data limitations, ensuring model transparency, harmonizing protocols, and fostering regulatory collaboration.

**Fig. 3:**
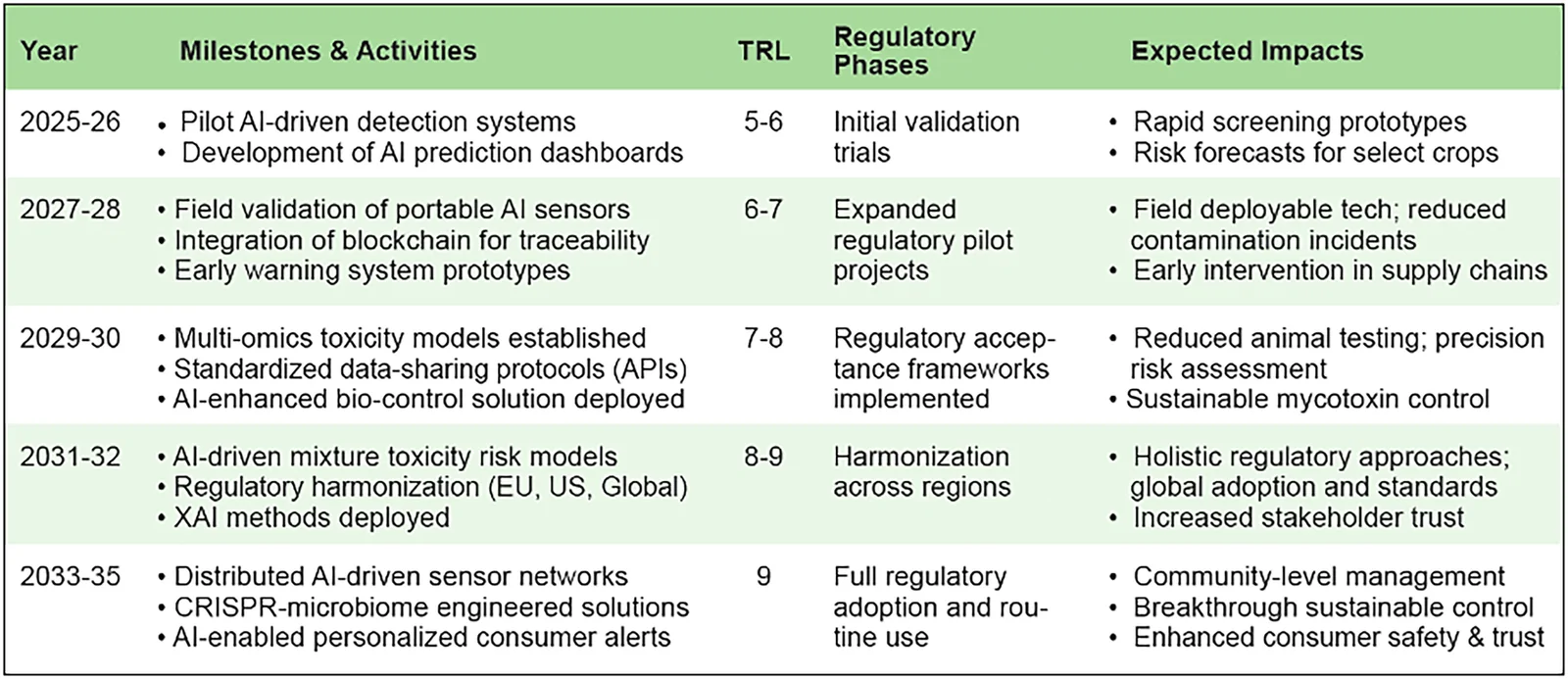
Future roadmap for AI in mycotoxin research and implementation. This roadmap illustrates a realistic, achievable pathway for advancing AI-driven solutions to significantly improve global mycotoxin management practices over the next decade. Technology Readiness Levels (TRLs): TRL 5-6: Technology validated in relevant environments; prototypes tested. TRL 6-7: Demonstrated in operational environments; system validation in field conditions. TRL 7-8: Full-scale demonstration; operational implementation and system refinement. TRL 8-9: Final form, system proven under expected conditions; ready for deployment. TRL 9: Fully mature technology, widely adopted in operational environments. Regulatory Phases: Initial validation trials: Preliminary evaluation, initial regulatory dialogues; expanded pilot projects: Broader-scale field validations, regulatory input on feasibility. Regulatory acceptance frameworks: Formal integration into existing regulatory guidelines; harmonization across regions: International agreements, aligned regulatory practices; full adoption & routine use: Standard regulatory procedures, widespread operational implementation. This roadmap illustrates a realistic, achievable pathway for advancing AI-driven solutions to significantly improve global mycotoxin management practices over the next decade. Expected Impacts: Enhanced food safety, Reduced contamination incidents, Minimized animal testing, Precision risk assessments, Increased sustainability in agricultural practices, Greater consumer safety and trust.

The integration of AI with multi-omics insights and mixture toxicity modeling will advance next-generation risk assessments, paving the way for truly precision-based mycotoxin control (Fig. 3). We call on researchers to prioritize interdisciplinary collaboration, industry leaders to invest in scalable implementations, and regulators to develop forward-thinking frameworks that accommodate AI innovation while ensuring safety standards. The benefits extend beyond mycotoxins to the broader food safety landscape, where AI-driven approaches could revolutionize how we address chemical and microbiological hazards.

## Supplementary information

Supplementary Information

## Data Availability

No datasets were generated or analysed during the current study.
