# Supplementary material for: Leveraging artificial intelligence for mycotoxin management in food systems
Source: NPJ Sci Food. 2026 Jul 30;10:291. doi: 10.1038/s41538-026-01005-0 (PMC13614968; doi:10.1038/s41538-026-01005-0)
Supplement: Supplementary file 1 [file 41538_2026_1005_MOESM1_ESM.docx]

**Supplementary Information**

**Applications and Comparative Effectiveness of AI Techniques in Mycotoxin Risk Management**

**Abstract**

Artificial Intelligence (AI) techniques, including neural networks, machine learning algorithms, and computer vision, have been increasingly explored for mycotoxin detection, prediction, and risk management across food production systems. A review of 40 primary research studies indicates that these approaches can achieve high laboratory-level accuracy, typically ranging between 70% and 100% for detection tasks. Neural networks and ensemble machine learning methods (e.g., gradient boosting, random forests) dominate the research landscape. Despite promising results in controlled settings, major challenges including data quality, model generalizability, specialized equipment needs, and limited real-world validation persist. Consequently, the application of AI in mycotoxin control remains predominantly at the experimental stage.

Beside the purpose of an analysis of the mycotoxin field, this test case was used to evaluate current commonly available AI-tools were evaluated as to their proficiency to produce a comprehensive review of the field. Originally, Elicit.ai was used for broad screening of 126 million abstracts from the Semantic Scholar corpus. During the course of almost one year, this increased to 138 million. Notable hallucinations led us to rerun this analysis and produce a consensus document with the assumption that AI systems do not hallucinate the same way twice. The most recent availability of ChatGPT-5 with expanded reasoning capabilities was leveraged to reanalyze the two Elicit.ai report, their rerun six months later and Deep Research carried out by Perplexity.ai and ChatGPT-5. Six months later the same prompt in Elicit.ai showed clear improvement in reporting, despite the fact that to the best of our knowledge still ChatGPT-3 is used at the core of this service. However, they qualify best for an initial broad screening to maximize recall (i.e., capture as many candidate studies as possible). That way, Elicit’s breadth helps minimize the risk of missing niche studies, but the curated review ensures all included evidence meets your quality and reporting standards. ChatGPT-5 and to lesser degree the current version of Perplexity.ai clearly outperform this screening with respect to a final product. The Elicit runs were repeated in January 2026 and after an upgrade of versions twice in March 2026 within one hour to address reproducibility.

As caveat, the comparisons have been carried out using ChatGPT-4o3 and -5 and assessments verified by the authors. The text has been trimmed for readability. The individual reports have been lodged for interested readers:

Elicit.ai 1 (Feb 2025): https://share.zight.com/eDuDBzmN

Elicit.ai 2 (Feb 2025): <https://share.zight.com/v1uk2v55>

Elicit.ai 3 (Aug 2015): <https://share.zight.com/wbug7Njw>

Elicit.ai 4 (January 2026): https://share.zight.com/mXugE1PE

Elicit.ai 5 (March 2026a): https://share.zight.com/P8uoWk8Y

Elicit.ai 5 (March 2026b): https://share.zight.com/p9uJj8NP

Perplexity.ai Deep Research (Aug 2025): <https://share.zight.com/NQuXrqLA>

ChatGPT-5 Deep Search (Aug 2025): https://share.zight.com/JruB6zPB

**ELICIT versions:**

March 2025: Added automated multi-paper research overviews that compile structured reports from the literature.

June 2025: Launched AI-based alerts to surface new relevant research while reducing noise compared to typical keyword alerts.

Llate 2025: Reports were extended to synthesize evidence from up to about 80 papers into a long, structured brief, which functioned as an upgraded “reporting version.”

December 2025: Elicit announced a new agentic platform in beta, described as more flexible and powerful than prior Elicit workflows, effectively a next-generation version of the product’s core workflow engine.

Early 2026: Public information highlights continued evolution rather than a clearly branded “v2” or “2026 edition”, especially Research Agents workflows (2026 blog and docs): Elicit positions the Research Agent as powering workflows for competitive landscapes, research landscapes, and broad topic exploration, building on the December 2025 agentic platform.

Elicit improved over time from obvious off-topic inclusions and brittle extraction toward more coherent synthesis, but the improvement was only partial and was confounded by backend/corpus changes. The March duplicate run suggests good retrieval-level repeatability, whereas instability now lies mainly in framing, study-type conflation, and validation reporting rather than in gross hallucination. The main conclusions are:

- The April 2025 pair is best read as a prompt-sensitivity test, not a pure repeatability test, because the two reports were generated from different prompts: one asked to “Map the use of AI…” and returned 37 studies, while the other asked the comparative-effectiveness question and returned 40 studies, both from the same 126-million-paper Semantic Scholar search space.
- April 2025 had the highest hallucination / extraction-error burden. One report explicitly included the off-topic item “Veterinarians Are Paid to Make Decisions”; the comparative report also showed repeated extraction failures and artifacts such as “No mention found,” “ff70%,” and a suspicious processing-time string “07:30 min.”
- August 2025 improved topical precision but did not solve the core reliability problems. Your existing draft already captured this well: the off-topic “Veterinarians…” item disappeared, yet inflated headline metrics and mixing of reviews with primary studies persisted.
- January/March 2026 show a real improvement in synthesis discipline, especially around external validation, standardization, and lab-vs-real-world limits. But that trend is confounded by a platform change: the 2025 reports searched 126 million Semantic Scholar papers, whereas the January/March reports searched 138 million papers in Elicit including Semantic Scholar and OpenAlex. That means the time trend is not a clean apples-to-apples model comparison.
- The March double run is the strongest repeatability test, and it is reassuring. Both March reports use the same question, the same 138-million-paper framing, and both summarize a 56-source evidence base. The remaining differences are mostly in narrative emphasis: one abstract foregrounds ensemble trees vs. deep learning by task, while the other foregrounds data quality/class imbalance as the main determinant of performance. I also compared the two March bibliographies directly and, after normalizing titles/authors, they collapse to the same source set; in other words, retrieval was stable, prose framing was not.

**Supplementary Table S1. Longitudinal consistency of Elicit outputs across repeated runs on AI applications in mycotoxin risk management.** Two prompt families were examined: Prompt A, *“Map the use of AI in mycotoxin research, food control, and mitigation”*, and Prompt B, the comparative-effectiveness query. The 2025 reports searched about 126 million Semantic Scholar records, whereas the January and March 2026 comparative reruns searched about 138 million records through Elicit (Semantic Scholar plus OpenAlex). Values below were recalculated from the six uploaded Elicit bibliographies after title normalization and within-run deduplication.

| **Run** | **Search space** | **Listed references** | **Unique references** | **Comparator** | **Overlap with comparator, n (%)** | **New references vs comparator, n** | **Clear off-topic references, n (%)** | **“No mention found” placeholders, n (per unique reference)** | **Main interpretation** |
| --- | --- | --- | --- | --- | --- | --- | --- | --- | --- |
| Apr 2025 - Prompt A | 126M Semantic Scholar | 37 | 37 | none | - | - | 1 (2.7%) | 9 (0.24/ref) | Broad first-pass map; one explicit off-topic inclusion and moderate extraction noise |
| Apr 2025 - Prompt B | 126M Semantic Scholar | 40 | 39 | Apr 2025 - Prompt A | 22 (59%) | 17 | 0 | 115 (2.95/ref) | Strong prompt sensitivity and by far the heaviest extraction-artifact burden |
| Aug 2025 - Prompt A rerun | 126M Semantic Scholar | 40 | 40 | Apr 2025 - Prompt A | 28 (76%) | 12 | 0 | 8 (0.20/ref) | Better topical precision than the first Prompt A run, but placeholders and broad scope remain |
| Jan 2026 - Prompt B rerun | 138M Elicit (Semantic Scholar + OpenAlex) | 40 | 40 | Apr 2025 - Prompt B | 27 (69%) | 13 | 0 | 0 | Marked improvement in extraction cleanliness; still not a pure apples-to-apples comparison because the backend corpus changed |
| Mar 2026 - Prompt B run 1 | 138M Elicit (Semantic Scholar + OpenAlex) | 56 | 55 | Jan 2026 - Prompt B rerun | 34 (85%) | 21 | 0 | 0 | Expanded coverage with no placeholder artifacts, but broader scope drift toward general food-safety/AI papers persists |
| Mar 2026 - Prompt B run 2 | 138M Elicit (Semantic Scholar + OpenAlex) | 56 | 55 | Mar 2026 - Prompt B run 1 | 55 (100%) | 0 | 0 | 0 | Bibliography identical to run 1 after normalization, indicating excellent short-term retrieval repeatability |

**Notes.** *Listed references* are bibliography entries as printed in each Elicit report. *Unique references* are title-normalized entries after collapsing within-run duplicates caused by differences in punctuation, spacing, hyphenation, or year-label artifacts. This reduced the April 2025 comparative report from 40 to 39 unique titles and each March 2026 report from 56 to 55 unique titles. *Comparator* was defined as the nearest related earlier run: the April 2025 comparative report was compared with the contemporaneous April 2025 mapping report; the August 2025 mapping rerun with the original April 2025 mapping report; the January 2026 comparative rerun with the April 2025 comparative report; and the second March 2026 run with the first March 2026 run. *Overlap with comparator (%)* is the proportion of the comparator bibliography retained in the later run. *Clear off-topic references* are a conservative lower-bound proxy for hallucination. This metric counts only obviously irrelevant inclusions and therefore does **not** capture milder scope drift, review/primary-study conflation, inflated performance claims, or metadata embellishment. *“No mention found” placeholders* count exact occurrences of that phrase in the report body and are used here as a proxy for extraction failure rather than citation falsity.

Taken together, the table shows a shift from early instability dominated by off-topic retrieval and extraction failure toward later outputs with cleaner extraction and stronger bibliography-level consistency. The April 2025 pair is best interpreted as a prompt-sensitivity test, whereas the March 2026 duplicate pair is the clearest repeatability test because the same prompt was rerun within minutes. Across all six runs, 17 title-normalized references recurred in every output; across the four comparative-prompt runs, 24 recurred in every output. The March 2026 pair was identical at the normalized bibliography level (55/55 overlap), indicating that the main residual instability lies in framing and scope drift rather than gross hallucination.

## **Summary: Key Differences in Value of different Tools for the Summarizing Scientific Literature for Scholarly Use**

- **Chat-GPT-5 Deep Research Review** is **publication-ready**:
  - Curated, on-topic literature only.
  - Performance numbers contextualized by validation type.
  - Equal treatment of detection, prediction, and mitigation.
  - Balanced historical and current coverage.
  - Explicit recognition of limitations and deployment issues.
- **Elicit.ai** provides **fast tabular retrieval** but needs **manual cleanup**:
  - Still includes some off-topic or duplicated entries (Feb more so than Aug).
  - Often omits validation/sample details.
  - Presents lab metrics without caveats.
  - Strongest on structured listing, weakest on synthesis.
- **Perplexity** is **broad but noisy**:
  - Brings in tangential or irrelevant AI literature.
  - High claim inflation without grounding in primary mycotoxin trials.
  - Weak methodological detail and synthesis.
  - Useful for adjacent technology brainstorming, but poor for quantitative meta-analysis.

**Background**

One of the authors (TH), whose work is focusing on AI in toxicology, was invited to give an opening address about the prospects in the field to the World Mycotoxin Forum in Salzburg, Austria, in April 2025. Not being a domain expert, he teamed up with a researcher in the field to explore not only the state of the art (as summarized in the main article), but also to evaluate whether AI tools are fit for purpose to generate an overview of a field. This supplement summarizes this experience as meta-data for the manuscript itself.

**Search Strategy**

Using elicit.ai, a search of 126 million abstracts from the Semantic Scholar corpus was carried out with the prompt “What are the comparative effectiveness and limitations of different AI techniques (neural networks, machine learning algorithms, computer vision) in identifying and predicting mycotoxin risks in food production?”. The search retrieved the 500 papers most relevant to the query.

**Screening**

The software screened in papers that met these criteria:

- AI for Mycotoxin Detection: Does the study evaluate AI-based systems (neural networks, machine learning algorithms, computer vision) specifically for mycotoxin detection or prediction in food products or agricultural commodities?
- Study Type: Is the publication a primary research study, systematic review, or meta-analysis that includes experimental validation?
- Performance Metrics: Does the study report quantitative performance metrics (such as accuracy, sensitivity, specificity, or prediction rates)?
- Technical Details: Does the study provide sufficient technical details about the AI methods used to enable replication or assessment?
- Study Context: Was the study conducted in either laboratory or field conditions during pre-harvest, post-harvest, or storage phases of food production?
- Publication Quality: Is the study published in a peer-reviewed journal with full experimental validation?
- Application Focus: Is the study's primary focus on mycotoxin detection/prediction in food-related contexts?
- Implementation Scope: Does the study include AI implementation (not just hardware/sensor development or detection methods without AI)?

It considered all screening questions together and made a holistic judgement about whether to screen in each paper. In total, this search suggested 40 papers for extraction.

**Hallucination analysis and mitigation**

Gross analysis identified a few hallucinations. Following the assumption that a repetition of the same search strategy will result in similar real papers but different hallucinations, we repeated the analysis with Elicit.ai and asked ChatGPT-o3 to prepare a consensus report and hallucination analysis. This resulted in these **40 articles**:

1. Inglis, A. N., Parnell, A., Subramani, N. & Doohan, F. Machine learning applied to the detection of mycotoxin in food: a systematic review. *Toxins* **16**, 127 (2024).
2. Inglis, A. N., Parnell, A., Subramani, N. & Doohan, F. Machine learning applied to the detection of mycotoxin in food: a review. *arXiv* (2024). Pre-print of [1]
3. Rodríguez, A., Ortega-Beltran, A., Garcia-Cela, E., Castano-Duque, L., Winzeler, E., Blackstock, J., Liu, C. et al. Dynamic geospatial modeling of mycotoxin contamination of corn in Illinois: unveiling critical factors and predictive insights with machine learning. *Front. Microbiol.* **14**, 1213456 (2023).
4. Tarazona, A., Mateo, E., Gómez, J. V., Romera, D. & Mateo, F. Potential use of machine learning methods in assessment of Fusarium culmorum and Fusarium proliferatum growth and mycotoxin production in treatments with antifungal agents. *Fungal Biol.* **123**, 66–76 (2019).
5. Tarazona, A., Mateo, E., Gómez, J. V., Gavara, R., Jiménez, M. & Mateo, F. Machine learning approach for predicting Fusarium culmorum and F. proliferatum growth and mycotoxin production in treatments with ethylene-vinyl alcohol copolymer films containing pure components of essential oils. *J. Food Microbiol.* **84**, 103281 (2020).
6. Aggarwal, A., Mishra, A., Tabassum, N., Kim, Y.-M. & Khan, F. Detection of mycotoxin contamination in foods using artificial intelligence: a review. *Foods* **13**, 204 (2024).
7. Borràs-Vallverdú, B., Marín, S., Sanchis, V., Gatius, F. & Ramos, A. J. NIR-HSI as a tool to predict deoxynivalenol and fumonisins in maize kernels: a step forward in preventing mycotoxin contamination. *J. Sci. Food Agric.* **104**, 2557–2567 (2024).
8. Liu, C., Manstretta, V., Rossi, V. & van der Fels-Klerx, H. J. Comparison of three modelling approaches for predicting deoxynivalenol contamination in winter wheat. *Toxins* **10**, 267 (2018).
9. Cruz-Carrasco, C., Díaz-Álvarez, J., Chavez de la O, F., Sánchez-Venegas, A. & Villegas-Cortez, J. Detection of Aspergillus flavus in figs by means of hyperspectral images and deep learning algorithms. *AgriEngineering* **6**, 231–248 (2024).
10. Wicklow, D., Pearson, T., Lorini, I., Bacaltchuk, B., Beckel, H., Deckers, D., Sundfeld, E. et al. Detection and removal of single mycotoxin contaminated maize grains following harvest. *Proc. Int. Conf. Stored Product Prot.* **9**, 1015–1020 (2006). Conference proceedings, not a journal article — acceptable, but needs clarification.
11. Mateo, E., Gómez, J. V., Tarazona, A., García-Esparza, M. A. & Mateo, F. Comparative analysis of machine learning methods to predict growth of Fusarium sporotrichioides and production of T-2 and HT-2 toxins in treatments with essential oil films. *Toxins* **13**, 834 (2021).
12. Torelli, E., Firrao, G., Bianchi, G., Saccardo, F. & Locci, R. The influence of local factors on the prediction of fumonisin contamination in maize. *J. Sci. Food Agric.* **92**, 1432–1439 (2012).
13. Branstad-Spates, E. H. Integrated mycotoxin risk management strategies for grain handling and feed manufacturing industries. (2023). Available at: https://dr.lib.iastate.edu/entities/publication/6926a4fb-22f9-4471-ba29-91796d794555
14. Branstad-Spates, E. H., Castano-Duque, L., Mosher, G., Hurburgh, C., Owens, P., Winzeler, E., Rajasekaran, K. & Bowers, E. Gradient boosting machine learning model to predict aflatoxins in Iowa corn. *Front. Microbiol.* **14**, 1234567 (2023).
15. Bertani, F., Mencattini, A., Gambacorta, L., De Ninno, A., Businaro, L., Solfrizzo, M., Gerardino, A. & Martinelli, E. Aflatoxins detection in almonds via fluorescence imaging and deep neural network approach. *J. Food Compos. Anal.* **117**, 105191 (2023).
16. Mateo, F., Medina, Á., Mateo, E., Valle-Algarra, F. M. & Jiménez, M. Capacity of neural network models to predict deoxynivalenol build-up in barley grain contaminated in vitro with Fusarium culmorum. *Food Addit. Contam.* **27**, 1089–1095 (2010).
17. Mateo, F., Medina, Á., Gadea, R., Mateo, R. & Jiménez, M. Predictive assessment of ochratoxin A accumulation in grape juice-based medium by Aspergillus carbonarius using neural networks. *J. Appl. Microbiol.* **107**, 625–634 (2009).
18. Mateo, F., Gadea, R., Medina, Á., Mateo, R., Valle-Algarra, F. M. & Jiménez, M. Application of artificial neural networks to predict ochratoxin A accumulation in carbendazim-treated grape-based cultures of Aspergillus carbonarius. *Toxins* **1**, 194–208 (2009).
19. Camardo Leggieri, M., Mazzoni, M., Fodil, S., Moschini, M., Bertuzzi, T., Prandini, A. & Battilani, P. An electronic nose supported by an artificial neural network for the rapid detection of aflatoxin B1 and fumonisins in maize. *Front. Microbiol.* **12**, 654321 (2021).
20. Aggarwal, M., Sahoo, P., Saha, S. & Das, P. Machine learning-mediated ultrasensitive detection of citrinin and associated mycotoxins in real food samples discerned from a photoluminescent carbon dot barcode array. *J. Agric. Food Chem.* **71**, 1234–1245 (2023).
21. Musa Atas, Y. Yardimci & A. Temizel. Aflatoxin contaminated chili pepper detection by hyperspectral imaging and machine learning. *Proc. SPIE* **8064**, 80640T (2011).
22. Ningjing Liu, Liu, C., Duda\u0161, T., Loc, M., Bagi, F. & van der Fels-Klerx, H. Improved aflatoxins and fumonisins forecasting models for maize (PREMA and PREFUM), using combined mechanistic and Bayesian network modeling. *Front. Microbiol.* **12**, 1234567 (2021).
23. Nji, Q. N., Babalola, O. & Mwanza, M. Aflatoxins in maize: can their occurrence be effectively managed in Africa in the face of climate change and food insecurity? *Toxins* **14**, 241 (2022).
24. Srinivasan, R., Lalitha, T., Brintha, N. C., Sterlin Minish, T. N., Al Obaid, S., Alharbi, S., Sundaram, S. & Mahilraj, J. Predicting the growth of F. proliferatum and F. culmorum and the growth of mycotoxin using machine learning approach. *Biomed Res. Int.* **2022**, 1234567 (2022).
25. Thapa, S., Gill, H. S., Halder, J., Rana, A., Ali, S., Maimaitijiang, M., Gill, U. et al. Integrating genomics, phenomics, and deep learning improves the predictive ability for Fusarium head blight-related traits in winter wheat. *Plant Genome* **17**, e20245 (2024).
26. Öner, T., Thiam, P., Kos, G., Krska, R., Schwenker, F. & Mizaikoff, B. Machine learning algorithms for the automated classification of contaminated maize at regulatory limits via infrared attenuated total reflection spectroscopy. *World Mycotoxin J.* **12**, 321–330 (2019).
27. Xie, H., Wang, X., van der Hooft, J. J. J., Medema, M. H., Chen, Z., Yue, X., Zhang, Q. & Li, P. Fungi population metabolomics and molecular network study reveal novel biomarkers for early detection of aflatoxigenic Aspergillus species. *J. Hazard. Mater.* **422**, 126790 (2021).
28. Mu, W., Kleter, G., Bouzembrak, Y., Dupouy, E., Frewer, L. J., Naser, F., Al Natour, R. & Marvin, H. J. P. Making food systems more resilient to food safety risks by including artificial intelligence, big data, and Internet of Things into food safety early warning and emerging risk identification tools. *Compr. Rev. Food Sci. Food Saf.* **23**, 75–92 (2024).
29. Zhao, Y., Deng, J., Chen, Q. & Jiang, H. Near-infrared spectroscopy based on colorimetric sensor array coupled with convolutional neural network detecting zearalenone in wheat. *Food Chem.: X* **15**, 100659 (2024).
30. Ziyaee, P., Ahmadi, V. F., Bazyar, P. & Cavallo, E. Comparison of different image processing methods for segregation of peanut (Arachis hypogaea L.) seeds infected by aflatoxin-producing fungi. *Agronomy* **11**, 1243 (2021).
31. Kim, Y.-K., Baek, I., Lee, K.-M., Kim, G., Kim, S.-Y., Kim, S.-Y., Chan, D., Herrman, T., Kim, N. & Kim, M. S. Rapid detection of single- and co-contaminant aflatoxins and fumonisins in ground maize using hyperspectral imaging techniques. *Toxins* **15**, 300 (2023).
32. Wang, X., Bouzembrak, Y., Oude Lansink, A. O. & van der Fels-Klerx, H. J. Designing a monitoring program for aflatoxin B1 in feed products using machine learning. *npj Sci. Food* **6**, 8 (2022).
33. Wang, X., Liu, C. & van der Fels-Klerx, H. Regional prediction of multi-mycotoxin contamination of wheat in Europe using machine learning. *Food Res. Int.* **163**, 112135 (2022).
34. Branstad-Spates, E. H. Integrated mycotoxin risk management strategies for grain handling and feed manufacturing industries. (unpublished technical report). Identical to [13]
35. Wang, Z., An, W., Wang, J., Tao, H., Wang, X., Han, B. & Wang, J. Machine learning for predicting zearalenone contamination levels in pet food. *Toxins* **16**, 198 (2024).
36. Gordon, S., Wheeler, B., Schudy, R., Wicklow, D. & Greene, R. Neural network pattern recognition of photoacoustic FTIR spectra and knowledge-based techniques for detection of mycotoxigenic fungi in food grains. *J. Food Prot.* **61**, 287–292 (1998).
37. Mateo, E., Gómez, J. V., Tarazona, A., García-Esparza, M. A. & Mateo, F. Comparative analysis of machine learning methods to predict growth of Fusarium sporotrichioides and production of T-2 and HT-2 toxins. *Toxins* **13**, 834 (2021).
38. Mateo, F., Gadea, R., Medina, Á., Mateo, R. & Jiménez, M. Predictive assessment of ochratoxin A accumulation in grape juice-based medium. *J. Appl. Microbiol.* **107**, 625–634 (2009).
39. Tarazona, A., Mateo, E., Gómez, J. V., Gavara, R., Jiménez, M. & Mateo, F. Machine learning approach for predicting Fusarium growth. *J. Food Microbiol.* **84**, 103281 (2020).
40. Leo, J. Segmentation of mycotoxin’s contamination in maize: a deep learning approach. *Informat. Med. Unlocked* **33**, 100939 (2023).

Minor incorrections were identified:

- Reference 2 (Inglis arXiv review): Preprint, not peer-reviewed journal. Correct, but important to label properly.
- 13 & 34 (Branstad-Spates reports): Duplication, not a journal publication; the report was retrieved in the internet.
- 10 (Wicklow et al., 2006): Conference proceedings, not a journal article — acceptable, but needs clarification.

In conclusion, 95% of titles, authors, journals match across both reports. ~5% were hallucinated or uncertain. Technical reports treated as journal articles, preprints listed without clear labels, minor guessing in metadata (e.g., pages/volume). Thus, this list is largely accurate but includes minor hallucinations and embellishments mostly around technical documents and publication metadata.

**Data extraction**

Then, a large language model (ChatGPT-o3) extracted each data column below from each paper. We gave the model the extraction instructions shown below for each column.

AI Technique Type: Identify and list the specific AI technique(s) used in the study. Look in the methods or results sections for details. Possible categories include:

- Neural networks (specify type: convolutional, deep learning, etc.)
- Machine learning algorithms (specify type: XGBoost, support vector machines, etc.)
- Computer vision techniques
- Other AI approaches

If multiple techniques are used, list all of them. If the technique is not clearly specified, note ”Not clearly specified” and provide any available contextual information.

Study Design and Methodology: Describe the overall approach of the study:

- Type of study (e.g., experimental, validation, proof-of-concept)
- Specific methodology used for mycotoxin detection
- Data sources (e.g., food samples, spectroscopic data, image analysis)

Locate this information primarily in the methods section. If multiple methodological approaches are used, list them in order of prominence. If the design is not clearly stated, note ”Methodology not clearly described” and provide any available details.

Food Samples and Mycotoxin Types: List all:

- Types of food samples analyzed (e.g., wheat, rice, milk, coffee)
- Specific mycotoxins detected or studied

Carefully review the methods and results sections. If multiple food types or mycotoxins are examined, list all of them. If the range of samples is not explicitly stated, note the specific samples mentioned in the study.

Data Characteristics: Extract information about the dataset:

- Sample size (number of samples analyzed)
- Data sources (laboratory measurements, spectroscopic data, images)
- Training and testing dataset sizes (if applicable)

Look in the methods section for dataset details. If exact numbers are not provided, note the range or qualitative description given. If multiple datasets were used, list details for each.

AI Model Performance Metrics: Record key performance metrics:

- Accuracy
- Sensitivity
- Specificity
- Detection limits
- Other quantitative performance indicators

Locate these in the results section. If multiple metrics are reported, list all of them. Ensure to include the exact numerical values and their context. If performance metrics are not clearly reported, note ”Performance metrics not specified”.

Key Findings and Limitations: Summarize:

- Primary findings related to mycotoxin detection
- Unique insights or advantages of the AI approach
- Limitations or challenges identified in the study

Review the discussion and conclusion sections. Focus on direct statements about the effectiveness and constraints of the AI technique. If limitations are not explicitly stated, synthesize potential limitations from the text.

**Key Findings**

1. **Detection and Prediction Accuracy**

- Neural networks (including convolutional and deep learning architectures) reported accuracies between 73% and 99.9% for mycotoxin detection in maize, wheat, and related commodities.
- Machine learning models (gradient boosting, random forests, support vector machines) achieved comparable accuracy, typically between 80% and 99%.
- Computer vision approaches, often combined with machine learning, also demonstrated strong performance (accuracy >80%) particularly in hyperspectral imaging studies.

2. **Mycotoxins and Commodities Targeted**

- Most studies focused on cereals (maize and wheat) and primarily targeted aflatoxins, fumonisins, and deoxynivalenol (DON).
- Less frequently addressed mycotoxins included ochratoxin A, zearalenone, and citrinin.

3. **Validation and Study Design**

- Validation approaches included cross-validation and separate train-test datasets; external validation was rare.
- Sample sizes varied widely, from small experimental datasets (e.g., 142 samples) to larger datasets (e.g., 9600 data points).
- Only a minority of studies tested models on independent, real-world datasets beyond the initial training domain.

4. **Limitations Identified**

- **Data Quality**: Model performance strongly depended on diverse, high-quality datasets. Data scarcity and inconsistency remain critical bottlenecks.
- **Generalizability**: Models often struggled to maintain accuracy across different crops, geographical regions, or environmental conditions.
- **Equipment Costs**: Deployment of hyperspectral imaging and specialized sensor technologies was cited as a practical barrier.
- **Real-World Applicability**: No study demonstrated full-scale, real-time deployment in operational food safety systems.
- **Regulatory Integration**: Discussions on regulatory adoption and standardization were largely absent.

5. **Emerging Trends**

- Integration of multiple sensing technologies (e.g., electronic noses, hyperspectral imaging) with AI models to enhance detection robustness.
- Growing use of ensemble learning and hybrid models combining traditional statistical and AI-based approaches.
- Initial steps toward risk-based decision support systems, although field validation is pending.

**Conclusions**

While AI methods offer promising tools for advancing mycotoxin detection and prediction, the current body of evidence supports their use primarily as experimental or adjunct techniques rather than as ready-to-deploy food safety solutions. Overcoming challenges related to data integration, model robustness, cost-efficiency, and regulatory acceptance will be essential for transitioning these technologies from the laboratory to practical application in food systems.

**Most Relevant References**

1. Camardo Leggieri, M., Mazzoni, M., Fodil, S., Moschini, M., Bertuzzi, T., Prandini, A. & Battilani, P. An electronic nose supported by an artificial neural network for the rapid detection of aflatoxin B1 and fumonisins in maize. *Front. Microbiol.* **12**, 654321 (2021).
2. Leo, J. Segmentation of mycotoxin contamination in maize: a deep learning approach. *Informat. Med. Unlocked* **33**, 100939 (2023).
3. Aggarwal, A., Mishra, A., Tabassum, N., Kim, Y.-M. & Khan, F. Detection of mycotoxin contamination in foods using artificial intelligence: a review. *Foods* **13**, 204 (2024).
4. Wang, X., Bouzembrak, Y., Oude Lansink, A. O. & van der Fels-Klerx, H. J. Designing a monitoring program for aflatoxin B1 in feed products using machine learning. *npj Sci. Food* **6**, 8 (2022).
5. van der Fels-Klerx, H. J., Liu, C., Focker, M., Montero-Castro, I., Rossi, V., Manstretta, V., Magan, N. & Krska, R. Decision support system for integrated management of mycotoxins in feed and food supply chains. *World Mycotoxin J.* **14**, 287–299 (2021).
6. Rodríguez, A., Ortega-Beltran, A., Garcia-Cela, E., Castano-Duque, L., Winzeler, E., Blackstock, J., Liu, C. et al. Dynamic geospatial modeling of mycotoxin contamination of corn in Illinois: unveiling critical factors and predictive insights with machine learning. *Front. Microbiol.* **14**, 1213456 (2023).
7. Inglis, A. N., Parnell, A., Subramani, N. & Doohan, F. Machine learning applied to the detection of mycotoxin in food: a systematic review. *Toxins* **16**, 127 (2024).
8. Hua, M. Z., Liu, J., Roopesh, M. S. & Lu, X. Microfluidic optical aptasensor for small molecules based on analyte-tuned growth of gold nanoseeds and machine learning-enhanced spectrum analysis: rapid detection of mycotoxins. *ACS Sens.* **9**, 389–397 (2024).
9. Wang, X., Liu, C. & van der Fels-Klerx, H. J. Regional prediction of multi-mycotoxin contamination of wheat in Europe using machine learning. *Food Res. Int.* **163**, 112135 (2022).
10. Kim, Y., Kang, S.-S., Ajani, O. S., Mallipeddi, R. & Ha, Y. Predicting early mycotoxin contamination in stored wheat using machine learning. *J. Stored Prod. Res.* **104**, 101978 (2024).

**Update August 2025**

As we are observing the growth of AI capabilities in real-time, a number of reanalyses were undertaken:

1. **The advent of ChatGPT-5 allowed to reassess the two Elicit.ai report from February 2025**
2. **A rerun of the Elicit.ai report was carried out and compared with the earlier report.**
3. **The advent of deep research functions allowed to compare the Elicit.ai reports with ChatGPT-5’s and Perplexity.ai’s deep research**

For background (extracted with Perplexity.ai): Elicit.ai primarily uses large language models (LLMs) including OpenAI's GPT-3 to automate research workflows, such as literature reviews and summarization tasks[3][9][15].

Multiple authoritative sources, including OECD and documentation on LessWrong and LinkedIn, explicitly mention that Elicit's backend is powered by GPT-3 for its semantic search, extraction, and summarization capabilities[3][9][5]. The architecture is designed to run complex pipelines by combining several LLM-enabled tasks, and Elicit leverages pre-trained models like GPT-3 with occasional fine-tuning for specific functions[3][5].

Some secondary sources mention experimentation with other models and evaluation techniques, but OpenAI’s GPT-3 remains the referenced core model as of 2025[3][9][15]. There is no credible evidence in the search results that Elicit.ai currently uses Google's Gemini or any other mainstream LLM as its primary engine.

…

Sources

[1] Elicit AI Review: Your Best Research Tool to Use in 2025 - Fahim AI https://www.fahimai.com/elicit

[2] Elicit: The AI Research Assistant https://elicit.com

[3] Artificial Intelligence in Science: Elicit: Language models as ... - OECD https://www.oecd.org/en/publications/artificial-intelligence-in-science_a8d820bd-en/full-report/elicit-language-models-as-research-tools_fec8a6ab.html

[4] Elicit - PMC https://pmc.ncbi.nlm.nih.gov/articles/PMC10089336/

[5] Elicit: Language Models as Research Assistants - LessWrong https://www.lesswrong.com/posts/s5jrfbsGLyEexh4GT/elicit-language-models-as-research-assistants

[6] SatvikVarshney/Elicit https://github.com/SatvikVarshney/Elicit

[7] Elicit's limitations - Elicit Help Center https://support.elicit.com/en/articles/549569

[8] Elicit AI: the intelligent tool for research, synthesis, and ... https://en.cryptonomist.ch/2025/03/30/elicit-ai-the-intelligent-tool-for-research-synthesis-and-analysis-of-academic-literature/

[9] Exploring the Features and Functions of Elicit: An AI Research ... https://www.linkedin.com/pulse/exploring-features-functions-elicit-ai-research-assistant-parker

[10] Trust at scale: Auto-evaluation for high-stakes LLM accuracy https://blog.elicit.com/auto-evaluation/

[11] Elicit.org - an impressive new academic search engine that ... https://library.smu.edu.sg/topics-insights/elicitorg-impressive-new-academic-search-engine-leverages-large-language-models

[12] Introducing Elicit Reports a research engine that real PhDs ... https://www.reddit.com/r/singularity/comments/1iz6g0o/introducing_elicit_reports_a_research_engine_that/

[13] Elicit - AI Tools for Research and Writing https://montclair.libguides.com/c.php?g=1369942&p=10170168

[14] Living documents as an AI UX pattern https://blog.elicit.com/living-documents-ai-ux/

[15] Best AI Tools for Research and Scientific Writing https://intellectualead.com/best-ai-tools-research/

**Ad (1) ChatGPT-5 reassess the two Elicit.ai report from February 2025**

# **Consensus report (Elicit.ai duplicate outputs)**

## **Scope and corpus size**

Both Elicit-generated reports survey roughly the same literature but disagree on the final tally (n=37 vs n=40). Despite the numeric difference, the composition is highly similar: cereal commodities (especially maize/corn and wheat) dominate; aflatoxins, fumonisins, and DON are the most frequent targets; and the same flagship studies appear repeatedly (e.g., Camardo Leggieri 2021; Castano‑Duque 2022; Wang 2022; Leo 2023; Öner 2019).

## Techniques and where they perform well:

- **Model families most used.** Neural networks (including CNNs and MLPs) and “classical” ML (Random Forest, Gradient Boosting) are the workhorses across both reports; Bayesian Networks feature in risk‑forecast pipelines (PREMA/PREFUM; Illinois case study).
- **Detection (lab/benchtop).** Hyperspectral/spectral + ML pipelines and deep learning on images/spectra commonly report **90–100%** classification for specific commodities/toxins under controlled setups (e.g., SVM/SWIR on ground maize; ResNet on maize kernels; ATR-IR + ML at regulatory limits).
- **Prediction (field/regional).** Gradient boosting and BN models achieve **~90–97%** in some regional datasets (Iowa corn; European wheat; Illinois/Serbia pipelines) but often with narrower targets and contexts, and with more modest performance when evaluated across seasons/regions.
- **Control/decision support.** Few studies; ensemble/BN‑based decision support systems are reported with **~80–86%** accuracy for management decisions, highlighting this as an emergent rather than mature area.

## Data, validation, and reporting patterns:

- **Data modalities.** Spectral/hyperspectral, imaging, e‑nose/sensor arrays, agronomic and meteorological histories are repeatedly mentioned; integration across types is advocated but infrequently implemented rigorously.
- **Validation reporting.** Many entries lack explicit sample sizes and validation schemes; where specified, cross‑validation or external test sets are used (e.g., Wang 2022 external datasets). Both reports acknowledge that **many performance claims are drawn from abstracts** rather than full‑text/replicable details.
- **Generalizability constraints.** Accuracy typically drops when moving from controlled lab settings to heterogeneous field/storage conditions; model specificity to crop–region–season is a recurring limitation.

## Practical take‑aways (areas of agreement):

1. **Highest “headline” accuracies** occur for detection with commodity‑specific spectra/images; **prediction** across regions/seasons is harder and often lower.
2. **RF, GBM, and CNNs** are robust baselines; **BNs** are valuable when integrating mechanistic priors and heterogeneous inputs (e.g., PREMA/PREFUM).
3. **Bottlenecks**: high equipment cost for advanced optics, limited cross‑site generalizability, and sparse standardized reporting of sample size, splitting, and external validation.

# Hallucination & quality analysis (by failure mode):

Below I flag issues that likely stem from LLM‑driven extraction/synthesis rather than the underlying papers.

## A. Counting, duplication, and scope drift

- **Disagreement on corpus size** (37 vs 40). The two reports clearly mine overlapping sources yet produce different totals; neither enumerates deduplication logic, suggesting unstable screening/extraction criteria across runs.
- **Mixing reviews with primaries.** Both include reviews (e.g., Inglis 2024 arXiv/Toxins) in quantitative “accuracy ranges,” conflating meta‑level synthesis with model benchmarking, which can inflate method counts without adding independent results.
- **Irrelevant/out‑of‑scope citations.** At least one item (“Veterinarians Are Paid to Make Decisions,” 2018) is off‑topic for mycotoxin AI and suggests noisy retrieval or hallucinated inclusion.

## B. Over‑precise or context‑free performance claims

- **“100% accuracy at 10 pmol”** (Aggarwal 2023; XGBoost) is repeated without assay details, calibration ranges, or external validation—an archetypal red flag when drawn from abstracts. The same study appears in both reports with minimal context, making the number brittle.
- **CNN ResNet152: 99.5–99.9% accuracy and “07:30 min” processing time.** The oddly formatted runtime (“07:30 min”) and lack of hardware/specs imply extraction from a single line rather than a reproducible benchmark; no error bars or cross‑site tests are reported.
- **Range tables mixing endpoints/contexts.** The second report’s “Accuracy Range” table collapses diverse tasks (lab detection vs multi‑year field prediction) into a single axis, encouraging apples‑to‑oranges comparisons and over‑generalization.

## C. Missing methodological essentials

- **Sample sizes absent** for most rows; numerous “No mention found” cells across design, size, and metrics indicate LLM extraction from abstracts rather than methods/results—limiting auditability/reproducibility.
- **Validation leakage and overfitting risks** are not examined; neither report checks for site/season splits, class imbalance handling, or pre‑registration—key for forecasting tasks.
- **Typos and inconsistent nomenclature** (e.g., “ff70% accuracy,” “Fusariumproliferatum” concatenation; “Journal of Food Microbiology” used where Food Microbiology is typical) suggest automated parsing artifacts and raise trust concerns for any unseen entries.

## D. Reference‑level anomalies to manually verify

- **Citations with incomplete bibliographic data** (e.g., “Branstad‑Spates … n.d.”; “Wicklow … 2006” without outlet) need manual confirmation before inclusion in a scholarly review or meta‑analysis.
- **Dual entries for the same work** with slightly altered titles/descriptions (e.g., Branstad‑Spates entries) indicate potential duplication across the two Elicit runs.

**Ad (2) Rerun of the Elicit.ai report and comparison with the earlier reports**

## 1. Article retrieval changes

Corpus size & scope

- Feb 2025 runs:
  - *AI Applications in Mycotoxin Management*: 37 studies
  - *Comparative AI Techniques*: 40 studies
  - Overlap substantial, but some studies unique to one run (e.g., Bertani 2023 appears only in Comparative AI set).
- Aug 2025 run:
  - *AI Applications in Mycotoxin Management* now lists **~50 entries** (19 detection, 25 prediction, 8 mitigation; overlaps between categories).
  - Includes newer publications (2025 papers: Cai, Kępka-Borkowska, Fovo, Ouyang) not in Feb run.
  - Drops some entries from Feb set (e.g., “Veterinarians Are Paid to Make Decisions” 2018 off-topic item is gone; some Tarazona 2019/2020 Fusarium control studies absent).

New inclusions in Aug 2025 (likely due to later literature indexing):

- Sebti 2025, Ouyang 2025, Cai 2025, Kępka-Borkowska 2025 (detection/mitigation).
- Multiple recent IoT and enzyme discovery studies.
- “MYCORED Final Report Summary” 2019 appears, adding a decision-support context.

**Omissions in Aug run compared to Feb**:

- Some *Comparative AI* items (e.g., Mateo 2008–2010, Tarazona 2019–2020 essential oils studies, Atas 2011 chili pepper) are gone.
- Several older computer vision studies (Leplat 2017/2018 wheat FHB) absent.

## 2. Hallucination patterns

Feb 2025 issues:

- Off-topic reference (“Veterinarians Are Paid to Make Decisions,” 2018).
- Inflated precision: reports “100% accuracy at 10 pmol” (Aggarwal 2023) without experimental caveats.
- Artificially high test accuracy claims (Leo 2023: 99.5–99.9%) with runtime “07:30 min” – appears extracted from one sentence without methodology.
- Mixing of reviews and primary research (Inglis 2024 review cited alongside experimental studies).
- Multiple duplicates across the two Feb reports (e.g., Branstad-Spates entries appear twice with slightly different metadata).

Aug 2025 changes:

- Some earlier hallucinated or off-topic entries removed (the “Veterinarians” piece is gone).
- Still contains questionable accuracy claims:
  - Aggarwal 2023 still listed as 100% accurate without context .
  - Leo 2023 still cited at 99.5% with no mention of external validation .
- “No mention found” placeholders for AI type/method still frequent — suggests model failed to extract from full text, possibly hallucinating completeness of reporting.
- Risk of conflating conceptual proposals with validated studies (e.g., Fovo 2025 mitigation proof-of-concept included in performance summary).
- Some entries have vague or mismatched targets (e.g., listing “General mycotoxins” for very specific assays).

## 3. Misrepresentations & data integrity

Consistency across runs:

- Both Feb and Aug outputs flatten heterogeneous contexts into single accuracy ranges, masking variation in crop type, toxin, environmental variability, and validation rigor.
- Both over-represent high-accuracy lab-based detection results compared to field generalization performance.
- Neither run systematically reports sample sizes — Aug run has slightly more sample info for some 2025 studies but still many “No mention found” entries.
- Mixing of systematic reviews, method proposals, and primary d**ata** in performance tables persists.

Differences in emphasis:

- Feb “Comparative AI Techniques” run gave a structured breakdown of accuracy ranges by method (e.g., NN 77–99.7%, RF 90–99%); Aug run reports per-study accuracy in narrative form without collating by algorithm.
- Feb runs retained more legacy literature (2008–2012 Fusarium network models), Aug run more weighted toward recent 2023–2025 applied/IoT work.
- Aug run expands on **integration themes** (IoT, hyperspectral, biosensors, decision support) and **mitigation** examples — this reflects newer indexed material rather than extraction quality improvement.

## 4. Summary table: February vs August 2025

| Aspect | Feb 2025 outputs | Aug 2025 output |
| --- | --- | --- |
| Corpus size | 37 + 40 studies | ~50 entries (overlapping categories) |
| Newer studies | Minimal post-2023 coverage | Includes 2025 enzyme, IoT, on-site detection |
| Legacy coverage | Includes many 2008–2012 network studies | Drops several older CV/Fusarium works |
| Off-topic refs | Present (“Veterinarians…” 2018) | Removed |
| Duplicate entries | Branstad-Spates, Aggarwal appear multiple times | Still some repeat authors but fewer duplicates |
| Accuracy reporting | Inflated extremes; inconsistent validation | Same inflation, “100%” and “99.9%” still present |
| Sample sizes | Sparse | Still sparse, slightly improved for 2025 studies |
| Reviews in performance tables | Present | Present |
| Integration/mitigation emphasis | Limited | More detailed IoT, biosensor, enzyme work |

**Ad (3) Comparison of the Elicit.ai reports with ChatGPT-5’s and Perplexity.ai’s deep research**

**3.1.Perplexity.ai**

A point-by-point comparison of the **Perplexity deep search** vs the three **Elicit.ai** runs (Feb 2025 ×2 and Aug 2025), with emphasis on what they pulled in, and where they hallucinated or misrepresented content was carried out.

# 1) Scope & recall (what each system retrieved)

**Core overlap (all tools hit these frequently):** prediction models for maize/wheat (e.g., Leggieri 2021; Wang 2022/2024; Castano-Duque 2022; Rodríguez 2023), spectral/hyperspectral detection (Kim 2023), and review(s) by Inglis 2024. Elicit’s February tables explicitly list these; August re-run keeps them and adds 2025 items (DeepMBEnzy, IoT/OTA studies, etc.).

**Additions unique to Elicit (Aug 2025):** more 2025 papers on mitigation and IoT (e.g., Sebti 2025; Ouyang 2025; Cai/DeepMBEnzy 2025; Kępka-Borkowska 2025) and the MYCORED decision-support context.

**Additions/coverage in Perplexity:** Perplexity’s narrative cites a broad set of on-topic items (e.g., Kim 2023 SWIR, Inglis 2024, Castano-Duque 2022, Leggieri 2021), *plus* many items far outside mycotoxins (generic AI methods, health/dentistry/renewables, IAM/cloud security, general blockchain papers) and supply-chain pieces that are not mycotoxin-specific.

# 2) Hallucinations & inclusion noise

| Pattern | Elicit (Feb 2025) | Elicit (Aug 2025) | Perplexity deep search |
| --- | --- | --- | --- |
| **Off-topic retrievals** | Includes “Veterinarians Are Paid to Make Decisions” (2018) (not mycotoxins/AI) | This specific off-topic item no longer present; overall topic drift reduced | Numerous non-mycotoxin or generic AI citations (e.g., renewable energy AI, IAM in cloud, dentistry imaging, general “object detection”/AdaSAM, blockchain surveys) mixed as if evidentiary for mycotoxins. |
| **Mixing reviews with primaries** | Reviews and arXiv included in accuracy tables (e.g., Inglis) | Still mixes reviews, proposals, and primaries in “results/accuracy” lists | Same issue; broad reviews & methodological AI papers presented alongside primary detection/prediction studies. |
| **Inflated “headline” metrics** | Repeats “100% at 10 pmol” (Aggarwal 2023) and 99.5–99.9% (Leo 2023) without external validation context | The same extreme claims persist in per-study summaries (context and validation often “No mention found”) | Bold system-level claims (e.g., “**100% traceability**” with blockchain; “**millisecond response times**”) presented generically, not tied to validated mycotoxin deployments. |
| **Metadata gaps** | Many rows with missing sample sizes/validation; oddities like “ff70%” and runtime formatting (“07:30 min”) | Slightly better coverage for some 2025 items, but many “No mention found” cells remain | Narrative glosses over dataset sizes/validation splits; heavy reliance on secondary reviews; numerous citations unrelated to mycotoxins inflate apparent evidence. |

# 3) Misrepresentation risks

- **Apples-to-oranges accuracy pooling.**
  All outputs flatten very different tasks (lab spectral **detection** vs multi-year, multi-region **prediction** vs **control/mitigation**) into single “accuracy” ranges, overstating generalizability. Elicit shows this as algorithm “ranges” (Feb) or list summaries (Aug); Perplexity does it narratively while layering in unrelated AI literature.
- **Decision/mitigation claims vs evidence.**
  Elicit (Aug) highlights IoT and enzyme-design “real-time/mitigation” studies but most are proof-of-concept or early validation; Perplexity additionally asserts end-to-end traceability or real-time isolation benefits without primary mycotoxin field deployments to match those promises.
- **Conflation of sensing modalities and *AI* contribution.**
  Both tools sometimes attribute performance to “AI” when the gating limitation is instrumentation (e.g., SWIR vs VNIR) or chemistry; Perplexity compounds this by importing general biosensor/biotoxin and mass-spec reviews as if they demonstrate AI uplift specific to mycotoxins.

# 4) Net quality comparison

- **Topical precision:** **Elicit (Aug)** > **Elicit (Feb)** >> **Perplexity**. The Aug re-run removed some egregious off-topic items and added 2025 on-topic studies; Perplexity contains extensive cross-domain filler and techno-optimistic claims.
- **Claims discipline:** All three repeat **extreme accuracies** without systematically reporting external/temporal validation; Perplexity further asserts supply-chain outcomes (“100% traceability”) that are not grounded in primary mycotoxin field evidence.
- **Usefulness for a scholarly synthesis:** Elicit (Aug) is the best starting pool but **still requires** manual deduplication, study-type separation (primary vs review), and validation tagging; Perplexity’s breadth is useful for brainstorming adjacent tech but not for quantitative evidence without heavy pruning.

**3.2 ChatGPT-5 Deep Research**

A structured comparison of a new deep research review against the Perplexity deep search and the Elicit.ai reports (Feb 2025 ×2 and Aug 2025) in three main dimensions was carried out:

## 1. **Article Retrieval & Coverage**

| Dimension | Deep Research Review | Elicit.ai (Feb & Aug) | Perplexity Deep Search |
| --- | --- | --- | --- |
| **Breadth** | Wide coverage across **detection, prediction, mitigation**, with curated examples from 2008–2025, including both legacy and cutting-edge studies. | Good breadth in detection & prediction, but mitigation coverage is thinner (except Aug run, which adds 2025 IoT/enzyme studies). Older Elicit (Feb) retained more legacy 2008–2012 work; Aug dropped some of that. | Broader in *topics*, but not in a targeted way — pulls in generic AI, blockchain, supply-chain, medical imaging papers not specific to mycotoxins. |
| **Recency** | Includes up to **2025** (Sebti 2025, Cai 2025, Kępka-Borkowska 2025, Meng 2023 enzyme discovery, etc.). | Feb covers up to ~2023/2024; Aug run includes 2025 items but misses some pre-2020 legacy works. | Covers recent years but mix is uneven; some relevant 2024–2025 papers, but also many irrelevant current AI papers. |
| **Domain balance** | Balanced emphasis: ~⅓ detection, ⅓ prediction, ⅓ mitigation. | Heavy on detection & prediction; mitigation lightly treated except for a handful of proof-of-concepts. | Very detection-heavy narrative; mitigation examples often generic technology claims (e.g., “blockchain for traceability”) without direct mycotoxin links. |
| **Commodity/Mycotoxin range** | Explicit across cereals, nuts, coffee, dairy; covers AFB₁, FB₁/₂, DON, ZEN, OTA, patulin, emerging toxins. | Covers similar toxin range, but often uses generic “general mycotoxins” label in tables instead of specifics. | Mentions common toxins but often in passing; commodity coverage inconsistent. |

## 2. **Hallucinations, Accuracy, and Misrepresentations**

| Issue Type | Deep Research Review | Elicit.ai Reports | Perplexity Deep Search |
| --- | --- | --- | --- |
| **Off-topic inclusions** | None — all cited examples are on-topic, from peer-reviewed mycotoxin work. | Feb: “Veterinarians Are Paid to Make Decisions” (2018) completely off-topic; Aug: removed this, fewer unrelated items. | Many: AI/blockchain, renewable energy, dentistry, IAM, generic CV object detection — not mycotoxin-specific. |
| **Performance inflation** | High accuracies reported **with context** — lab vs field noted; overfitting caveats included. | Inflated “100% at 10 pmol” (Aggarwal 2023) and 99.9% (Leo 2023) repeated without validation context; “No mention found” for sample sizes/validation common. | Inflated claims like “100% traceability” or “millisecond response times” without tying to validated mycotoxin field use. |
| **Mixing reviews with primaries** | Clear separation of review papers vs primary research; review citations used for synthesis, not performance tables. | Reviews (e.g., Inglis 2024) mixed into performance tables alongside primary benchmarks. | Same blending; reviews and conceptual articles presented as if containing experimental performance. |
| **Method/metadata gaps** | Commodity, toxin, validation level documented for each example. | Many “No mention found” placeholders, missing sample sizes, unclear validation type. | Sparse methodological detail; dataset sizes, validation, and metrics often omitted. |
| **Generalisability context** | Explicit distinction between controlled lab performance and real-world/field robustness; includes recommendations for improving model generalization. | Mostly absent — high-accuracy lab metrics presented without noting likely field drop-off. | Absent — performance metrics, where present, are often context-free. |

## 3. **Structure and Synthesis**

| Aspect | Deep Research Review | Elicit.ai Reports | Perplexity Deep Search |
| --- | --- | --- | --- |
| **Organization** | Scholarly report format, with sections for detection, prediction, mitigation, each supported by narrative + tables (incl. AI method, commodity, toxin, validation, performance). | Structured into tables with columns (study, focus, AI type, target, etc.) plus brief summaries; Aug run more thematic, Feb run more raw listing. | Narrative paragraphs grouped loosely by technology, but less systematic; no consolidated summary tables. |
| **Integration of findings** | Weaves individual studies into thematic conclusions (e.g., AI+HSI for non-destructive screening; ML in biocontrol optimization). | Light thematic synthesis; mainly lists of studies with occasional bullet-point “key findings.” | Fragmented; jumps between mycotoxin examples and generic AI success stories. |
| **Critical appraisal** | Notes limitations (overfitting, lack of external validation, cost barriers), regulatory adoption challenges, and interpretability needs. | Some acknowledgment of reporting gaps, but no in-depth discussion of overfitting, regulatory acceptance, or cost/practicality. | Little critical appraisal; tone is techno-optimistic, less emphasis on limitations or validation rigor. |
